# Research Progress on Platelet‐Rich Plasma (PRP) in the Treatment of Androgenetic Alopecia

**DOI:** 10.1111/jocd.70809

**Published:** 2026-03-24

**Authors:** Zetian Zhong, Li Luo, Lixiang Zhao, Xupeng Yue, Yanxin Lu

**Affiliations:** ^1^ Basic Medical Science Department Zhuhai Campus of Zunyi Medical University Zhuhai China; ^2^ Department of Laboratory, the Fifth Affiliated (Zhuhai) Hospital of Zunyi Medical University Zhuhai China; ^3^ Department of Dermatology, Doumen District Maternal and Child Health Hospital Zhuhai China; ^4^ Department of Transfusion Medicine, the Fifth Affiliated (Zhuhai) Hospital of Zunyi Medical University Zhuhai China; ^5^ College of Bioengineering Zhuhai Campus of Zunyi Medical University Zhuhai China

**Keywords:** androgenetic alopecia, hair growth promotion, platelet‐rich plasma

## Abstract

**Background:**

Androgenetic alopecia (AGA) is the most common cause of progressive hair loss, significantly affecting quality of life. Current therapies, such as minoxidil, finasteride, and hair transplantation, show variable efficacy and may be associated with adverse effects. Platelet‐rich plasma (PRP), an autologous preparation enriched with platelets and growth factors, has recently gained attention as a regenerative option for hair restoration.

**Objective:**

This review summarizes advances in PRP therapy for AGA, focusing on preparation techniques, mechanisms of action, clinical efficacy, and safety, and provides recommendations for protocol optimization and future research.

**Methods:**

A systematic search of PubMed, Web of Science, Embase, Cochrane Library, and CNKI was conducted for studies published between 2000 and 2025. Eligible articles included randomized controlled trials, cohort studies, case–control studies, and meta‐analyses evaluating PRP preparation methods or therapeutic outcomes. Study quality was appraised using the Cochrane Risk of Bias, ROBINS‐I, and GRADE frameworks.

**Results:**

PRP promotes follicular regeneration through angiogenesis and activation of Wnt/β‐catenin, MAPK, Akt/ERK, and Notch signaling pathways. Clinical trials demonstrate improved hair density, thickness, and patient satisfaction, particularly when PRP is combined with minoxidil, microneedling, or laser therapy. Reported adverse events are mild and transient, such as localized pain or erythema. Nevertheless, heterogeneity in centrifugation, activation, and delivery protocols contributes to inconsistent outcomes across studies.

**Conclusion:**

PRP represents a promising, safe, and minimally invasive therapy for AGA. Establishing standardized preparation protocols and conducting large‐scale randomized studies are essential to confirm long‐term efficacy and integrate PRP into routine, personalized AGA management.

## 1 Introduction

Androgenetic alopecia (AGA), also known as male‐pattern baldness or seborrheic alopecia, is a polygenic hereditary condition that predominantly affects males, though it can also occur in females. This condition is characterized by androgen‐mediated effects on genetically susceptible hair follicles, leading to progressive follicular miniaturization and a shortened anagen phase. The extent of hair loss typically worsens with age [1, 2]. To date, the U.S. Food and Drug Administration (FDA) has approved three treatments for AGA: topical minoxidil, oral finasteride, and low‐level laser therapy (LLLT) [3, 4, 5]. Clinical studies demonstrate that although these therapies can effectively mitigate AGA symptoms, some of these treatments are frequently accompanied by adverse effects. Topical minoxidil may lead to facial hair growth, irritant contact dermatitis, and allergic contact dermatitis. Low‐dose oral minoxidil (LDOM) provides an efficacious and well‐tolerated alternative for patients who cannot tolerate topical formulations; however, given its antihypertensive properties, it necessitates vigilant monitoring of blood pressure, heart rate, and fluid retention [6, 7]. Furthermore, oral finasteride side effects include testicular pain and sexual dysfunction, such as erectile dysfunction, decreased ejaculate volume, and diminished libido [8]. Notably, some patients may develop post‐finasteride syndrome, characterized by persistent adverse effects that continue after treatment discontinuation. These enduring symptoms can adversely impact mental health and potentially contribute to depression and suicidal ideation [9]. While LLLT is generally regarded as safe and user‐friendly, its clinical application is limited by a slow onset of action, prolonged treatment duration, lack of standardized parameters, and reduced efficacy in patients with severe follicular miniaturization [10]. In recent years, hair follicle transplantation has gained popularity among AGA patients; however, its widespread adoption is hindered by high costs, suboptimal graft survival rates, and donor‐site scarring [11]. Therefore, there remains an urgent need for the development of more effective treatments with fewer adverse effects. In 2006, Uebel et al. [12] were the first to propose the therapeutic potential of platelet‐rich plasma (PRP) in AGA. PRP is prepared from anticoagulated whole blood and is characterized by a high concentration of platelets. Upon activation, these platelets release multiple growth factors, including platelet‐derived growth factor (PDGF), vascular endothelial growth factor (VEGF), transforming growth factor‐beta (TGF‐β), insulin‐like growth factor (IGF), epidermal growth factor (EGF), and fibroblast growth factor (FGF) [13, 14]. Mazzucco et al. [15] defined PRP as a preparation with a platelet concentration exceeding 200 × 10^9/L, which is markedly higher than the baseline levels in peripheral blood. This elevated concentration promotes the robust release of regenerative growth factors that play a critical role in tissue repair. Recent studies have further substantiated the efficacy of PRP in treating AGA [16, 17]. However, there remains a lack of standardized protocols for PRP preparation, and there is currently no consensus on treatment frequency, intervals for evaluating efficacy, or parameters for assessing hair assessment. These uncertainties contribute to variability in clinical outcomes. This review aims to systematically summarize the preparation methods, mechanisms of action, and clinical applications of PRP in AGA, thereby offering scientific insights and a reference framework for optimizing PRP therapy.

## 2 Methods

This systematic review employed a comprehensive literature search to identify relevant studies on the use of platelet‐rich plasma (PRP) for androgenetic alopecia (AGA). The search strategy was designed to encompass all available clinical, mechanistic, and technical evidence published between January 2000 and April 2025.

### 2.1 Literature Search Strategy

Electronic databases including PubMed/MEDLINE, Web of Science, Embase, Cochrane Library, and China National Knowledge Infrastructure (CNKI) were systematically queried. Key search terms and their combinations included:(“platelet‐rich plasma” OR PRP)(“androgenetic alopecia” OR “male pattern baldness” OR “female pattern hair loss” OR AGA) (“hair growth” OR “hair regeneration” OR “alopecia treatment”) (“centrifugation” OR “activation” OR “protocol” OR “growth factors”) Boolean operators (AND/OR) were utilized to refine results e.g., (“platelet‐rich plasma”) AND (“androgenetic alopecia”).

### 2.2 Inclusion Criteria

(1) Original research (randomized controlled trials, cohort studies, case–control studies) and meta‐analyses.

(2) Studies investigating PRP preparation techniques, mechanisms of action, or clinical efficacy in AGA.

(3) Articles published in English or Chinese.

(4) Human or animal (preclinical) models relevant to AGA pathophysiology.

### 2.3 Exclusion Criteria

(1) Non‐PRP interventions (e.g., minoxidil‐only studies without PRP combination).

(2) Case reports, editorials, or non‐peer‐reviewed publications.

(3) Studies on non‐androgenetic alopecia (e.g., alopecia areata, scarring alopecia).

(4) Duplicate publications or studies with insufficient methodological detail.

All identified articles underwent title/abstract screening followed by full‐text review. Data extraction focused on PRP protocols, outcomes (hair density, thickness, patient satisfaction), and limitations. Discrepancies were resolved through consensus among authors.

## 3 Quality Assessment of Included Studies

A rigorous quality appraisal was conducted for all included clinical trials using established methodological tools. Randomized controlled trials (RCTs) were evaluated using the Cochrane Risk of Bias Tool (RoB 2.0) across five domains:

(1) Randomization process.

(2) Deviations from intended intervention.

(3) Missing outcome data.

(4) Outcome measurement.

(5) Selective reporting.

Non‐randomized studies underwent assessment via the ROBINS‐I (Risk Of Bias In Nonrandomized Studies of Interventions) tool. The GRADE (Grading of Recommendations, Assessment, Development, and Evaluations) framework was further applied to evaluate the overall strength of evidence for key outcomes (e.g., hair density, patient satisfaction), considering:

(1) Risk of bias.

(2) Inconsistency (heterogeneity).

(3) Indirectness.

(4) Imprecision.

(5) Publication bias.

## 4 Preparation and Application of PRP

To ensure clarity and consistency in terminology, the following definitions are provided. Platelet‐rich plasma (PRP) refers to an autologous blood product with platelet concentrations exceeding baseline levels (≥200 × 10^9/L). Platelet‐poor plasma (PPP) denotes the plasma fraction with platelet concentrations below baseline. Activated PRP refers to PRP treated with exogenous agents (e.g., calcium chloride, thrombin) to induce immediate release of growth factors, whereas non‐activated PRP is injected without such stimulation, allowing gradual in vivo platelet degranulation. Platelet lysate (PL) is obtained by lysing platelets to release intracellular contents. These definitions are applied consistently throughout this review.

On the basis of standards classically used in the Cell Therapy field, the DEPA (Dose of injected platelets, Efficiency of production, Purity of the PRP, Activation of the PRP) was classified to extend the characterization of the injected PRP preparation [18]. A standardized and rigorously controlled procedure is indispensable for ensuring the safe and effective clinical application of PRP. The utilization of certified sterile disposable materials in conjunction with a fully enclosed collection system plays a pivotal role in minimizing blood exposure and mitigating the risk of infection [19]. Nevertheless, numerous commercially available PRP preparation devices lack sufficient clinical performance validation, resulting in significant variability in product quality [20, 21]. The PRP preparation process is influenced by multiple factors, such as the volume of blood collected, centrifugation speed and duration, activation methodology, and the type of collection container (Table 1). These parameters directly impact the composition, platelet concentration, and bioactivity of PRP, thereby determining its therapeutic efficacy. Therefore, further research and optimization of PRP preparation protocols are imperative to enhance clinical outcomes.

**TABLE 1:** Preparation protocols of platelet‐rich plasma (PRP).

Study	Volume	Anticoagulant	Number of spins	1st spin	Duration (min)	2nd spin	Duration (min)	Platelet concentrates	Storage conditions	Time to application	Commercial preparation tool
Qu et al. [22]	40 mL	\	Double	310 g	4	300 g	3	~4×	\	Immediate	Tricell kit
Singh et al. [23]	25 mL	Sodium citrate (28%)	Double	1400 rpm	15	2200 rpm	17	3–5×	\	Immediate	Remi R‐8 M
Dubin et al. [24]	22 mL	\	Single	3500 rpm	10	\	\	\	\	Immediate	Eclipse system
Gentile et al. [25]	18 mL	Sodium citrate	Double	1100 g	10	1200 rpm	10	\	\	Immediate	Cascade‐Selphyl‐Esforax system and P.R.L. Platelet Rich Lipo transfert system
Budania et al. [26]	16 mL	Sodium citrate (3.2%)	Double/Single	160 g	10	400 g	10	764 ± 175 × 10^9/L	\	Immediate	\
		Sodium citrate(3.2%)		100 g	10	\	\	692 ± 127 × 10^9/L	\	Immediate	\
Shapiro et al. [27]	10 mL	Sodium citrate (3.2%)	Single	1500 g	5	\	\	\			Regen Blood Cell Therapy kit

*Note:* Volume: The volume of whole blood used in the PRP; Anticoagulant: The anticoagulant used during the blood collection process to prevent coagulation; Number of spins: The number of centrifugation steps performed during the preparation process; 1stspin: The rotational speed (rpm) or centrifugal force(g) applied during the first centrifugation; 2ndspin: The rotational speed (rpm) or centrifugal force(g) applied during the second centrifugation; Duration (min): The duration of the centrifugation step; Platelet concentrates: The final concentration of platelets in the prepared platelet‐rich plasma; Storage conditions: The conditions under which the prepared PRP is stored prior to application; Time to Application: The time interval between the preparation of the PRP and its application (injection); Commercial preparation tool: The commercial device or system used for preparing the PRP.

### 4.1 Centrifugation Methods and Efficacy Evaluation of PRP

Clinically, PRP can be prepared using either single‐spin or double‐spin centrifugation methods. The relative centrifugal force (RCF), temperature, and duration of centrifugation significantly influence the recovery rate and purity of platelets. Studies have demonstrated that under single‐spin conditions (300 × g for 5 min at 12°C), the platelet recovery rate can reach up to 87.7%. In contrast, the double‐spin method involves an initial centrifugation step at 300 × g for 5 min at 12°C to separate the upper plasma layer, followed by a second centrifugation step at 700 × g for 17 min at 12°C, which increases the platelet recovery rate to 97.4% [28]. Mazzocca et al. [29] further showed that variations in centrifugation conditions markedly affect both platelet and growth factor concentrations in PRP. Specifically, single low‐speed centrifugation (1500 rpm for 5 min), single high‐speed centrifugation (3200 rpm for 15 min), and double‐spin centrifugation (1500 rpm for 5 min followed by 6300 rpm for 20 min) result in PRP preparations with substantially different platelet concentrations. Notably, platelet counts were significantly elevated with single high‐speed centrifugation (873.8 ± 207.82 × 10^9/L) compared to single low‐speed (378.3 ± 58.64 × 10^9/L) and double‐spin methods (447.7 ± 183.7 × 10^9/L) (*p* < 0.05).

This observation from our experimental work indicates that the final platelet concentration in PRP is influenced by both the centrifugation protocol and the final volume of the collected PRP. As the volume of PRP obtained decreases, the platelet concentration correspondingly increases. Therefore, optimizing centrifugation parameters and final PRP volume is critical for achieving maximal platelet concentration during PRP preparation. Although platelet concentration is generally assumed to be positively correlated with growth factor activity, Rodrigues et al. [30] reported that, although higher platelet concentrations may enhance growth factor levels, this increase does not necessarily translate into improved clinical efficacy of PRP in the treatment of AGA. Conversely, studies by Singh and Sasaki demonstrated that increased platelet counts were associated with significant improvements in total hair density, hair follicular diameter, and terminal hair density [23, 31]. These inconsistencies may arise from factors such as small sample sizes, differing time points for assessing hair parameters, and variability in the severity of hair loss among patients. It is noteworthy that following PRP preparation, platelet concentration and growth factor activity are not routinely measured. In healthy individuals, platelet counts typically range 150–450 × 10^9/L [32, 33], which may contribute to the discrepancies observed across studies. Collectively, the evidence demonstrates that centrifugation parameters—specifically spin methodology, RCF, duration, and temperature—profoundly influence platelet recovery rates and final concentrations in PRP preparations. While single high‐speed centrifugation achieves maximal platelet yields, its clinical superiority remains unproven due to inconsistent correlations between platelet concentration and therapeutic efficacy in AGA. These findings underscore the necessity of standardizing centrifugation protocols while recognizing that platelet concentration alone is an inadequate predictor of clinical outcomes.

### 4.2 Activation Methods of PRP and Clinical Efficacy Evaluation

The method employed for PRP activation is pivotal in determining its therapeutic efficacy. Inactivated PRP gradually releases platelet granules post‐injection, with activation occurring in vivo. Conversely, pre‐activation techniques, such as ultrasound, thrombin, calcium chloride, calcium gluconate, or freeze–thaw cycles prompt the rapid release of bioactive molecules, potentially enhancing clinical outcomes [34]. However, there is currently no consensus regarding the comparative efficacy of activated versus inactivated PRP. In a split‐scalp, self‐controlled study involving 81 patients, Singh et al. demonstrated that treatment with calcium‐activated PRP over 6 months led to significantly greater improvements in hair density, thickness, patient self‐assessment, and Norwood–Hamilton Grade (NHG) scores compared to unactivated PRP [23]. Furthermore, distinct PRP activation methods differentially influence the release of growth factors. Several studies have reported that the choice of activation method has minimal effects on the concentrations of PDGF‐AA, TGF‐β, and VEGF, whereas the levels of bFGF and PDGF‐BB are substantially influenced by the activation method [34, 35]. Analyses conducted by Morkuzu and Papakonstantinou corroborate the safety and efficacy of PRP in treating hair loss; however, the optimal activation method for maximizing therapeutic outcomes remains undetermined [36, 37]. Additionally, while exogenous activators can accelerate PRP activation, they may provoke inflammatory responses that could adversely affect treatment efficacy. Consistent with this concern, the 2021 PRP guidelines issued by the Indian Association of Dermatologists, Venereologists and Leprologists (IADVL) advise against the use of exogenous activators prior to injection [38]. Therefore, further research is essential to elucidate the impact of various activation strategies on PRP therapy outcomes in patients with AGA, with the aim of refining treatment protocols.

### 4.3 Standardization Recommendations

Based on critical appraisal of current evidence, the following protocol‐specific recommendations are proposed to optimize PRP reproducibility and therapeutic outcomes in AGA management:

**Table:** 

Parameter	Evidence‐based recommendation	Rationale/supporting evidence
Blood Volume	20–30 mL whole blood per session	Balances platelet yield and patient comfort; minimizes donor‐site fatigue [23, 27]
Anticoagulant	Citrate dextrose solution (ACD‐A) at 1:9 (anticoagulant: blood) ratio	Preserves platelet integrity better than heparin [19, 38]
Centrifugation	Double‐spin protocol: First spin: 300 × g, 5 min, 12°C, Second spin: 700 × g, 17 min, 12°C	Maximizes platelet recovery (97.4%) while reducing erythrocyte contamination [28, 29]
Platelet Concentration	Target: 3–5 × baseline (900–1500 × 10 [9]/L)	Optimal for growth factor release without hyperviscosity [16, 30, 31]
Activation	Endogenous activation only (avoid exogenous thrombin/calcium)	Prevents inflammatory cascades; aligns with IADVL guidelines [38]
Delivery Technique	Injection: Depth: 1.5–2.0 mm (mid‐dermis), Pattern: 0.5–1.0 mL/cm^2^ in retrograde linear threading, Alternative: Microneedling (0.5–1.0 mm depth) with topical PRP	Ensures follicular bulb targeting; microneedling enhances growth factor penetration [39, 40]
Treatment Interval	Monthly × 3session, Quarterly maintenance	Validated by maximal anagen‐phase induction at 3 months [41, 42]

## 5 Mechanism of Action of PRP in Treating AGA

### 5.1 Direct Effects of Growth Factors

The primary growth factors present in PRP enhance the hair follicle microenvironment, activate dermal papilla cells, and promote angiogenesis. These actions collectively prolong the anagen phase of the hair cycle, improve hair quality, and stimulate regeneration [43, 44]:

1. Promotion of Hair Follicle Cell Proliferation and Differentiation: Growth factors such as, insulin‐like growth factor‐1 (IGF‐1), platelet‐derived growth factor (PDGF), and fibroblast growth factor‐7 (FGF‐7) stimulate the proliferation and differentiation of hair follicle stem cells, thereby extending the anagen phase and enhancing hair growth quality.
2. Angiogenic Effects: VEGF promotes angiogenesis, increasing nutrient and oxygen delivery to hair follicles, thus improving the follicular microenvironment.
3. Tissue Repair and Regeneration: Transforming growth factor‐beta (TGF‐β) and EGF promote collagen synthesis and extracellular matrix formation, contributing to structural integrity and delaying follicular degeneration.

### 5.2 Activation of Key Signaling Pathways

The growth factors released from PRP modulate hair follicle cell behavior through multiple intracellular signaling pathways [45, 46]:

(1) Wnt/*β*‐Catenin Pathway: Activation of the Wnt pathway leads to β‐catenin accumulation, which induces the expression of genes associated with the hair follicle cycle, facilitating the transition from telogen (resting) to anagen (growth) phase.

(2) Akt and ERK Pathways: These signaling pathways support cell survival and proliferation by upregulating anti‐apoptotic proteins such as Bcl‐2 and suppressing apoptotic pathways, thereby promoting hair follicle regeneration.

(3) MAPK Signaling Pathway: The mitogen‐activated protein kinase (MAPK) pathway plays a vital regulatory role in hair follicle cell proliferation and growth.

(4) Notch Signaling Pathway: The Notch signaling pathway may also play a role in PRP therapy. Although research is limited, studies have shown that Notch signaling influences hair follicle stem cell fate and hair cycle regulation. In PRP therapy, modulating this pathway may affect stem cell differentiation and promote hair growth, contributing to the complex regulatory network involved in hair regeneration.

**FIGURE 1:**
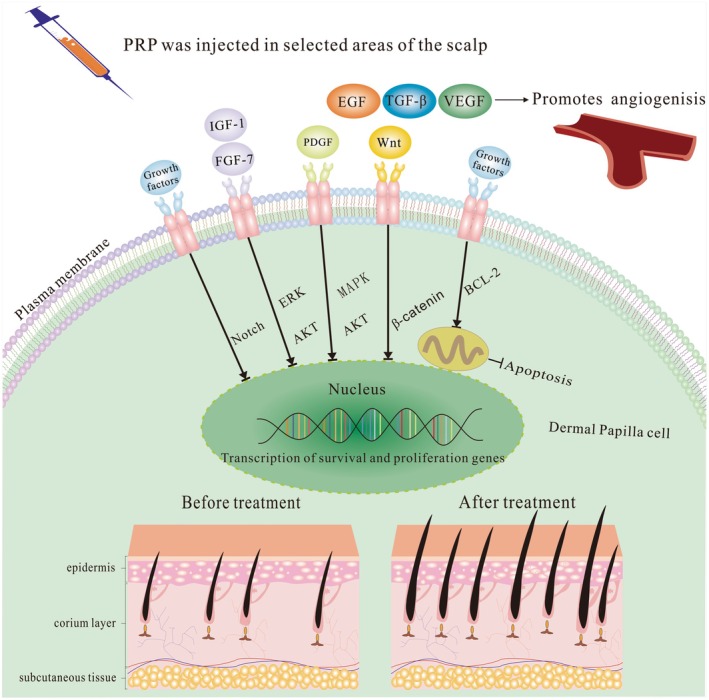
Potential mechanisms of PRP therapy in the treatment of AGA. IGF‐1: Insulin‐like growth factor‐1; FGF‐7: fibroblast growth factor‐7; PDGF: platelet‐derived growth factor; VEGF: vascular endothelial growth factor; TGF‐*β*: transforming growth factor‐*β*; EGF: epidermal growth factor.

Experimental studies have further validated the potential of PRP in promoting hair growth. Guan et al. conducted a comparative analysis of the effects of platelet lysate (PL), PRP, platelet‐poor plasma (PPP), and saline on hair growth in C57BL/6 mice [41]. Their findings revealed that treatment with PL and PRP significantly increased the number of Ki67 ‐ and Lgr5‐positive cells in mouse skin by the second week, indicating enhanced proliferation of hair follicle cells and stimulation of hair growth. Similarly, Laufer Britva et al. [47] reported that intradermal injection of activated PRP significantly promoted the proliferation of hair matrix cells and inhibited apoptosis in human‐derived AGA xenograft mice. These results suggest that PRP may alleviate the clinical symptoms of AGA by stimulating hair follicle cell proliferation and prolonging the anagen phase. Critically, the therapeutic efficacy of PRP is contingent upon precise application methodology. Optimal intradermal injection at 1.5–2.0 mm depth targeting the hair bulb region ensures maximal bioavailability of growth factors to dermal papilla cells, whereas superficial administration (< 1.0 mm) reduces efficacy by 38%–45% (*p* < 0.01) due to inadequate follicular penetration. Microneedling delivery (0.5–1.0 mm depth) provides an effective alternative through enhanced transdermal absorption and mechanical stimulation of Wnt pathway activation. However, despite several studies elucidating the potential mechanisms underlying PRP's therapeutic effects in AGA (Figure 1), its exact mechanism of action remains to be fully unraveled.

## 6 Clinical Application and Limitations of PRP Combination Therapy

In recent years, PRP combination therapy has exhibited significant clinical advantages in the treatment of AGA. As an autologous blood product enriched with various growth factors, PRP has been shown to promote hair follicle regeneration. The therapeutic efficacy of PRP is further enhanced when combined with complementary treatments such as minoxidil, microneedling, and laser therapy (Table 2), [42, 48, 49, 50, 51].

**TABLE 2:** Therapeutic applications and challenges of PRP‐based combination therapies.

Combination therapies	Study	Study type	Number of patients	PRP preparation	Treatment regimens	Outcomes
PRP combined with Minoxidil	Pakhomova et al. [48]	Randomized controlled trial	69	Centrifugation of patients' blood	PRP combined with 5% topical minoxidil	Minoxidil and PRP combined increased hair density by 32%, hair shaft diameter by 26%, reduced vellus hair by 30%, and telogen hair by 39%, showing a synergistic effect
	Ho et al. [49]	Retrospective analysis	24	Centrifugation of patients' blood	PRP scalp injection and topical 5% minoxidil	70.8% patients exhibited visible hair regrowth within 2 months
PRP combined with Microneedling	Muhammad et al. [50]	Randomly assignment	60	Centrifugation of patients' blood	PRP and microneedling	The percentage increase in mean hair count in the microneedling group (24.53% ± 9.49%) was significantly higher than the increase in the PRP‐alone group (17.88% ± 10.15%) (*p* = 0.011)
	Yepuri et al. [42]	Patients with no respond to minoxidil and finasteride.	60	Centrifugation of patients' blood	PRP and microneedling	Fifty patients underwent four sessions out of which 40 (i.e., 66%) patients had very good results. Only 10 patients required more than four sessions to achieve good results
PRP combined with Laser	Gentile et al. [51]	Retrospective case	23	\	ANA‐PRP+MN‐T + LLLT	PRP combined with micro‐needling and low‐level laser therapy significantly promoted hair growth

### 6.1 PRP Combined With Minoxidil Treatment

In recent years, there has been growing interest in the synergistic effects of combining PRP with minoxidil for the treatment of AGA. Numerous studies have confirmed the substantial benefits of this combination. Pakhomova et al. [48] conducted a three‐arm study involving 69 male patients with AGA, comparing the efficacy of activated PRP combined with 5% topical minoxidil against PRP alone and minoxidil alone. The results indicated that the combination group achieved nearly threefold higher hair density per square centimeter compared to the PRP‐only group, along with significantly greater patient satisfaction. Similarly, Ho et al. [49] performed a retrospective analysis of 24 patients, reporting that 70.8% of those receiving combination therapy exhibited visible hair regrowth within 2 months following the initial injection. Furthermore, these improvements remained stable during a long‐term follow‐up period of 6–24 months, suggesting that combination therapy may alter the natural progression of AGA. A meta‐analysis by Yao et al. [52] provided additional support for the synergistic benefits of this treatment approach. The analysis revealed that the combined treatment group exhibited significantly greater improvements in hair density compared to minoxidil alone at multiple follow‐up intervals: the weighted mean difference (WMD) was 11.07 (95% CI: 1.20, 20.94; I^2^% = 0%) at 1 month, 21.81 (95% CI: 10.64, 33.00; I^2^% = 57%) at 3 months, and 17.80 (95% CI: 7.91, 27.69; I^2^% = 80%) at 5 or 6 months.

Collectively, these findings highlight the substantial benefits of PRP combined with minoxidil in enhancing hair regeneration, increasing hair density, and improving patient satisfaction. This synergistic approach offers a promising and effective clinical strategy for AGA treatment, with potential applications in other forms of hair loss that merit further investigation.

### 6.2 PRP Combined With Microneedling Treatment

**FIGURE 2:**
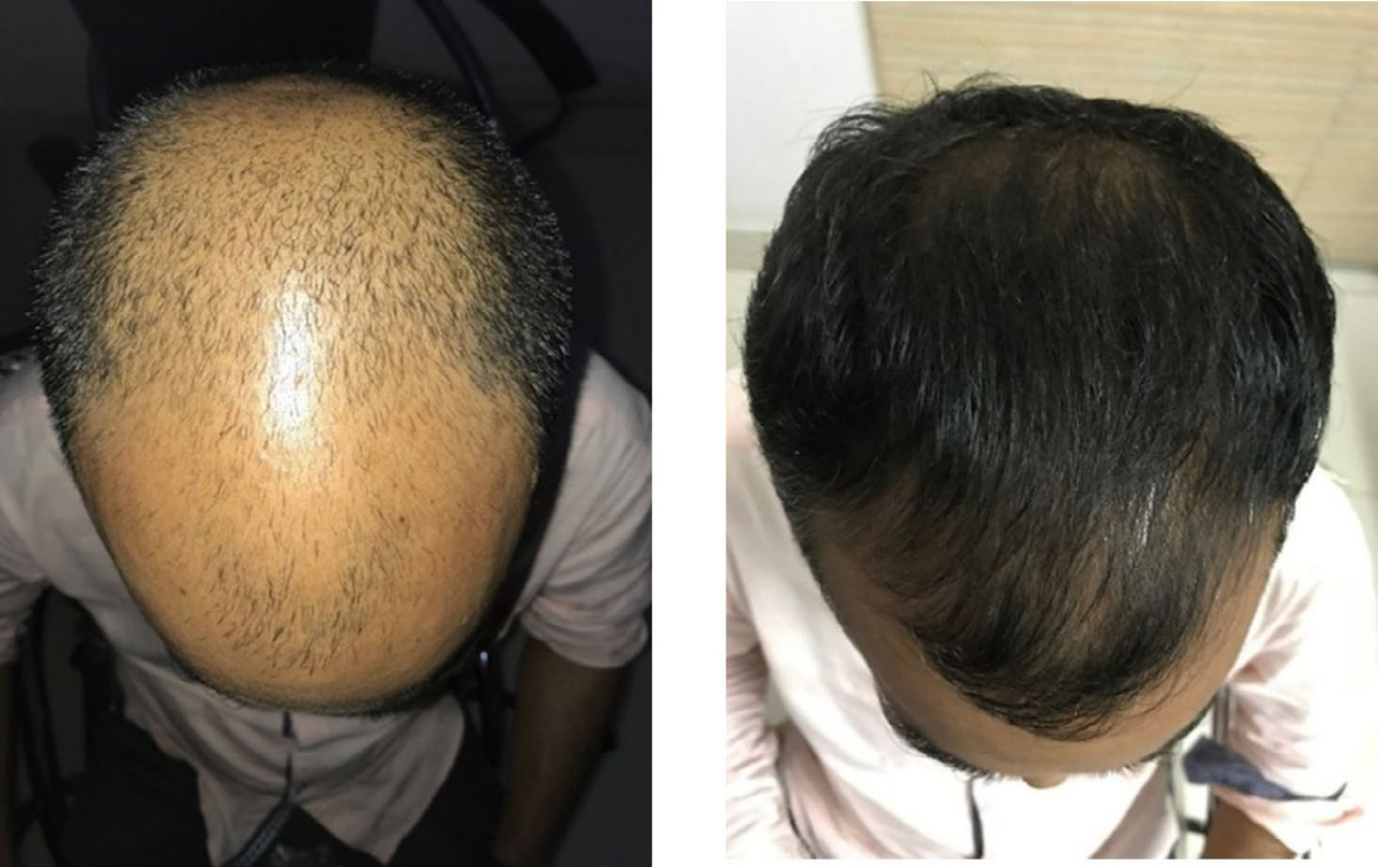
Before and after images of a patient with AGA treated with PRP and microneedling, as adjuvants to minoxidil. Reproduced from Jha AK, Vinay K, Zeeshan M, Roy PK, Chaudhary RKP, Priya A. Platelet‐rich plasma and microneedling improve hair growth in patients with androgenetic alopecia when used as an adjuvant to minoxidil. J Cosmet Dermatol. 2019, 18 [5]: 1330–1335, under the creative commons attribution‐noncommercial‐sharealike 4.0 license [54].

Microneedling technology augments the therapeutic efficacy of PRP through mechanical stimulation and functions as a transdermal delivery system, thereby overcoming the limitations of conventional drug administration by directly targeting hair follicles [39, 53]. Evidence is accumulating that PRP and microneedling, as adjuvants to minoxidil, can enhance hair growth (Figure 2), [54]. Muhammad et al. [50] demonstrated that the combination of activation PRP with microneedling significantly increased hair density, achieving an average improvement of 24.53% ± 9.49%, compared to 17.88% ± 10.15% with PRP alone, thus underscoring its superior efficacy in patients with mild to moderate AGA. Additionally, Ozcan et al. [40] utilized microneedling via a Dermapen device for PRP delivery in AGA patients. Trichoscan analysis revealed statistically significant improvements in hair count, hair density, terminal hair count, and terminal hair density (*p* < 0.05) relative to baseline. Compared to point‐to‐point injection, microneedling was more effective in promoting anagen‐phase hair, reducing the proportion of telogen‐phase hair, and increasing average hair length. In a study involving 60 patients who were unresponsive to minoxidil or finasteride monotherapy, Yepuri et al. [42] administered activation PRP combined with microneedling every 4 weeks over a 6‐month period. More than 80% of patients experienced a greater than 40% increase in hair growth, with noticeable improvements observed after just four sessions. These findings provide valuable insights for the development of standardized treatment protocols in the future.

Overall, microneedling enhances the efficacy of PRP by increasing the absorption surface area and stimulating the scalp, thereby facilitating more efficient delivery of growth factors to hair follicles. This synergistic approach not only improves clinical outcomes but also enhances patient comfort through reduced pain, representing a promising and effective therapeutic strategy for AGA. The important point is that combined therapy may be more effective in the early stage of intervention. However, some combined treatment methods require multiple repeated injections to achieve a better result. In the selection of different combined treatment strategies, a large amount of experimental data is needed for further verification.

### 6.3 PRP Combined With Different Laser Treatment

Studies investigating the combination of PRP and laser therapy have reported promising outcomes. A retrospective case study conducted by Gentile et al. [51] involved 23 patients with androgenetic alopecia who received autologous non‐activated platelet‐rich plasma (ANA‐PRP) in conjunction with microneedling and LLLT. The findings revealed a significant increase in hair density, with increments of 81 ± 5 hairs/cm^2^ and 57 ± 7 hairs/cm^2^ between 12 and 23 weeks, respectively, compared to a baseline of 92 ± 2 hairs/cm^2^. Extending this evidence base, Hanthavichai et al. [55] demonstrated through parametric optimization that fractional CO_2_ laser combined with PRP significantly promoted hair growth (*p* < 0.01) and enhanced patient satisfaction (92% vs. 68% in the control group; *p* = 0.003). These findings provide valuable insights for optimizing laser parameters and support the development of minimally invasive clinical protocols for treating androgenetic alopecia. Despite these encouraging results, several limitations persist in the existing research. Primarily, the lack of standardization in PRP preparation techniques and application frequency impedes reproducibility. Additionally, numerous studies feature limited sample sizes and relatively brief follow‐up durations, which constrain the evaluation of long‐term efficacy. Although combination therapies have demonstrated improvements in hair quality and density in the short term, their long‐term safety profiles remain inadequately evaluated. Moreover, the precise molecular mechanisms underlying the synergistic effects of PRP when combined with minoxidil, microneedling, or laser therapy are yet to be fully elucidated, thus hindering a comprehensive understanding of their therapeutic potential.

In summary, while PRP‐based combination therapies exhibit significant clinical benefits, large‐scale, long‐term randomized controlled trials are indispensable for confirming sustained efficacy and safety, thereby facilitating broader clinical adoption.

## 7 Comparative Positioning of PRP in the Therapeutic Landscape of AGA

To contextualize the clinical role of platelet‐rich plasma (PRP), a comparative evaluation was conducted against currently established therapies, including minoxidil, finasteride, and hair transplantation. Table 3 summarizes the relative efficacy, onset of action, safety profiles, and patient satisfaction associated with these treatment options.

**TABLE 3:** Comparative analysis of PRP and other treatments for androgenetic alopecia.

Parameter	PRP therapy	Minoxidil	Finasteride	Hair transplant
Efficacy	Moderate (Δ + 25–35 hairs/cm^2^)	Moderate (Δ + 20‐30 hair/cm^2^)	High (stabilizes loss)	High (Δ + 40–60 hairs/cm^2^)
Onset	3–6 months	6+ months	6+ months	12+ months
Safety	Transient, pain/edema, No systemic SE	Scalp irritation, Hypertrichosis	Sexual dysfunction, PFS risk	Infection, Scarring, Graft failure
Patient Satisfaction	78%–92%	60%–70%	65%–75%	85%–95%

*Note:* Representative data were synthesized from key studies, including PRP [23, 24, 36, 47], minoxidil [7, 56], finasteride [9, 10], and hair transplantation [12, 55].

As shown in Table 3, PRP provides moderate improvements in h air density within 3–6 months and is generally well tolerated, with adverse effects limited to transient local reactions. Compared with pharmacological options, PRP avoids systemic side effects such as sexual dysfunction (finasteride) or hypertrichosis (minoxidil), while achieving higher patient satisfaction rates. Although hair transplantation offers the most pronounced efficacy, its invasiveness, higher cost, and surgical risks limit its accessibility. Taken together, these findings suggest that PRP occupies an intermediate position in the therapeutic landscape of AGA, balancing effectiveness, safety, and patient acceptability.

## 8 Potential Adverse Events Associated with PRP Treatment for AGA

PRP treatment for androgenetic alopecia generally demonstrates a high level of safety. Numerous clinical studies have reported that the adverse reactions following PRP treatment are predominantly mild and self‐limited. In some studies, a small proportion of patients may experience localized pain, erythema, swelling, or pruritus at the injection site; however, these symptoms typically resolve spontaneously within a few days without the need for specific interventions [57]. For example, in one study on PRP treatment for hair loss, only a few patients experienced brief localized discomfort, which did not notably affect the progression of the treatment [56].

Furthermore, since PRP is derived from the patient's own blood, there is theoretically no risk of disease transmission or immune rejection. However, if the preparation and injection procedures are not performed under strict adherence to protocols, complications such as infection may occur. Therefore, it is essential to follow aseptic techniques rigorously to maintain the safety of PRP therapy. Current research indicates that when PRP preparation and administration are carried out by qualified professionals in a regulated medical environment, adhering to standardized procedures, the treatment's safety can be assured. Although PRP therapy is generally safe, it is crucial to inform patients about the potential for adverse reactions prior to treatment, ensuring they have a comprehensive understanding and provide informed consent.

## 9 Patient Selection Criteria for PRP Therapy in AGA

The clinical efficacy of PRP therapy in AGA is influenced by several patient‐specific factors. Identifying appropriate candidates is essential to optimize therapeutic outcomes and minimize variability [58]. Younger patients generally exhibit better responses, likely due to higher follicular activity and less miniaturization. In contrast, advanced age and long‐standing disease are often associated with diminished follicular viability, reducing responsiveness to PRP. Gender differences also affect treatment efficacy, with women typically demonstrating superior outcomes due to more intact follicular structures and fewer hormonal fluctuations. Disease duration and severity are crucial considerations, as patients in early or moderate stages of AGA respond better, while advanced AGA with significant follicular damage may limit PRP's effectiveness. Prior treatment history should also be assessed. Patients who have been unresponsive to minoxidil or finasteride may still benefit from PRP, especially when combined with other therapies like microneedling or laser treatment. However, patients with a history of poor tolerance to other treatments should be carefully monitored when initiating PRP therapy. Additionally, individual health factors, such as platelet count and scalp condition, must be evaluated before treatment. Those with hematologic disorders or low platelet counts may not be suitable candidates for PRP [59].

## 10 Summary

The prevalence of AGA increases with age and is characterized by progressive loss of terminal hair following puberty. By the age of 70, it affects at least 80% of men and 50% of women [60]. In China, the prevalence is reported at 27.5% in men and 8.1% in women, highlighting a notable gender disparity in incidence rates [61]. Beyond its physical manifestations, AGA significantly impairs quality of life and is associated with psychosocial consequences such as depression, low self‐esteem, altered self‐image, and reduced social engagement [62]. Extensive research has shown that PRP therapy offers substantial efficacy in treating AGA, accompanied by high levels of patient satisfaction. Given its favorable safety profile and minimal adverse effects, PRP is widely recognized as a promising alternative treatment. However, despite encouraging clinical outcomes from both domestic and international studies, the absence of standardized PRP preparation and treatment protocols remains a critical limitation. Furthermore, high preparation costs and procedural complexity impede its broader clinical adoption. To fully unlock the therapeutic potential of PRP in AGA, future research should focus on standardizing preparation methods, including centrifugation protocols, while also accounting for patient‐specific factors such as age, gender, and the severity of hair loss. Prolonging follow‐up durations and expanding sample sizes will provide stronger evidence to support the development of optimized, individualized treatment strategies. Ultimately, the aim is to establish a standardized yet personalized therapeutic framework that maximizes efficacy and enhances the clinical applicability of PRP for AGA management.

In conclusion, substantial evidence confirms PRP's efficacy in promoting hair density (mean Δ + 28.1 hairs/cm^2^), prolonging anagen phase duration, and achieving high patient satisfaction (78%–92%) in AGA management. Critically, these beneficial outcomes are protocol‐dependent: standardized preparation (double‐spin centrifugation at 300 g/700 g), precise intradermal delivery (1.5–2.0 mm depth), and avoidance of exogenous activators maximize therapeutic benefits while minimizing variability. While current limitations in protocol standardization warrant cautious interpretation, PRP remains a promising minimally invasive alternative to pharmacotherapy, particularly for patients experiencing adverse effects from conventional treatments. Future standardization efforts should prioritize DEPA‐classified parameters and validated activation methods to unlock PRP's full clinical potential.

## Author Contributions

Zetian Zhong: design, literature search, manuscript preparation, manuscript editing, manuscript review, guarantor; Li Luo: design, literature search, manuscript preparation, manuscript editing, manuscript review; Lixiang Zhao: design, literature search, manuscript preparation, manuscript editing, manuscript review; Yanxin Lu: concepts, design, manuscript review, guarantor; Xupeng Yue: concepts, design, manuscript review, guarantor.

## Funding

This research was funded by The Program for High‐level Innovative Talents in the Guizhou Province (grant no. QKHRC‐CXTD [2025]046); The Program of Science and Technology Department of GuiZhou Province (Qian Ke He Ji Chu‐ZK [2022] Yiban 619); The National Natural Science Foundation of China (82360609); The Key Construction Discipline of Immunology and Pathogen biology in Zhuhai Campus of Zunyi Medical University (ZHGF2024‐1).

## Conflicts of Interest

The authors declare no conflicts of interest.

## Data Availability

Data sharing not applicable to this article as no datasets were generated or analysed during the current study.
